# Allergen Immunotherapy (AIT): a prototype of Precision Medicine

**DOI:** 10.1186/s40413-015-0079-7

**Published:** 2015-11-10

**Authors:** GW Canonica, C. Bachert, P. Hellings, D. Ryan, E. Valovirta, M. Wickman, O. De Beaumont, J. Bousquet

**Affiliations:** Allergy and Respiratory Diseases -DIMI Department of Internal Medicine, University of Genova, IRCCS AOU San Martino, Genova, 16132 Italy; Upper Airways Research Laboratory, ENT-Department, University of Ghent, 9000 Ghent, Belgium; Department of Otorhinolaryngology, University of Leuven, Leuven, Belgium; Department of Otorhinolaryngology, University of Amsterdam, Amsterdam, The Netherlands; Allergy and Respiratory Research Group, Centre for Population Health Sciences, University of Edinburgh, Edinburgh, EH8 9AG UK; Department of Pulmonary and Allergic Diseases and Clinical Allergology, University of Turku, Turku, Finland; Department of Environmental Medecine, Karolinska Institutet, Sachs’ Children’s Hospital, Stockholm, Sweden; Stallergenes, Antony, France; University hospital, Montpellier, MACVIA-LR, Contre les Maladies Chronique spour un Vieillissement Actif en Languedoc Roussilon, European Innovation Partnership on Active and Healthy Ageing Reference Site, Montpellier, France; INSERM, VIMA : Ageing and chronic diseases Epidemiological and public health approaches, U1168 Paris, France; UVSQ, UMR-S 1168, Université Versailles St-Quentin-en-Yvelines, Versailles, France

**Keywords:** Allergen immunotherapy, AIT, Precision medicine, Personalized medicine, Allergy

## Abstract

Precision medicine is a medical model aiming to deliver customised healthcare - with medical decisions, practices, and/or products tailored to the individual patient informed but not directed by guidelines. Allergen immunotherapy has unique immunological rationale, since the approach is tailored to the specific IgE spectrum of an individual and modifies the natural course of the disease as it has a persistent efficacy after completion of treatment. In this perspective Allergen Immunotherapy - AIT has to be presently considered a prototype of Precision Medicine.

Precise information and biomarkers provided by systems medicine and network medicine will address the discovery of Allergen immunotherapy biomarkers for (i) identification of the causes, (ii) stratification of eligible patients for AIT and (iii) the assessment of AIT efficacy.

This area of medical technology is evolving rapidly and, compelemented by e-health, will change the way we practice medicine. It will help to monitor patients’ disease control and data for (i) patient stratification, (ii) clinical trials, (iii) monitoring the efficacy and safety of targeted therapies which are critical for reaching an appropriate reimbursement. Biomarkers associated with e-health combined with a clinical decision support system (CDSS) will change the scope of Allergen immunotherapy.

The cost/effectiveness of Allergen immunotherapy is a key issue for successful implementation. It should include the long-term benefits in the pharmaco-economic evaluation, since no other allergy treatment has this specific characteristic.

AIT is the prototype of current and future precision medicine.

## Introduction

**Fig. 1:**
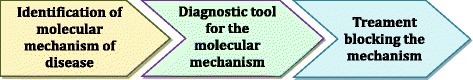
The three steps of personalised (precision) medicine (modified from [2])

Several terms including “personalized medicine”, “precision medicine”, “stratified medicine”, “targeted medicine” and “pharmacogenomics” are used interchangeably [1] but differ subtly. “Precision medicine” is similar to “personalized medicine” and is a new term encompassing one of the foremost examples of future disruptive innovation in healthcare. It is a medical model aiming at the customization of healthcare - with medical decisions, practices, and/or products tailored to the individual patient. It also refers to the tailoring of medical treatment to the individual characteristics of each patient [1]. In this model, based on the knowledge of mechanisms of the disease, personalized medicine generally involves the use of two medical domains, typically a diagnostic process and a therapeutic product, to select appropriate and optimal management, and to improve patients’ outcomes [2, 3] (Fig. 1). The term “personalized medicine” was first coined in the context of genetics, though it has since broadened to encompass many types of personalization measures.

“Stratification” refers to the division of patients with a specific disease into subgroups based on a particular characteristic and who respond more frequently (or better) to a treatment or alternatively are at decreased risk of side effects in response to a certain treatment.

The concept of precision medicine is not new: clinicians have long observed that patients with similar symptoms may have different illnesses, with different causes, and similarly that medical interventions may work well in some patients with the same disease. What is new is that advances in a wide range of fields from genomics, other omic sciences, to medical imaging to regenerative medicine, along with increased computational power and the advent of mobile and wireless capability and other technologies are allowing patients to be treated and monitored more precisely and effectively in ways that better meet their individual needs [1]. One of the recent examples is cystic fibrosis: the detection of the causative molecular mechanism of the disease in 4 % of the patients permitted a specific intervention which led to a complete reversion of the disorder [4]. The real and practical relevance in our society of precision medicine was highlighted by a new initiative launched in January 2015 by President Obama for cancer and diabetes mainly [5].

Precision medicine will cause the end of the blockbuster era in pharmaceuticals through better insights about the mechanisms of the diseases, the application of stratification in medicine, focussed research on drug safety and a model for financial return [6] (Table 1). There is an urgent need to adopt phenotype-driven therapy especially when expensive drugs such as biologics or biosimilars are prescribed [6]. This is fundamental to the sustainability of Health Care Spending.

**Table 1:** Major achievements of precision medicine

• Improve clinical outcomes and predictability	
• Avoid side effects caused by inappropriate treatment	
• Increase quality of life	
• Encourage patient compliance due to better results	
• Optimise use of healthcare resources	

**Fig. 2:**
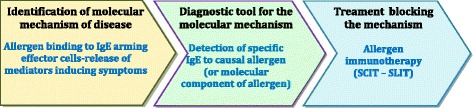
The three steps of Personalised (Precision) Medicine applied to AIT

AIT is a (current) (Table 2) and (future) (Table 3) paradigm of Precision Medicine [7]. It involves a precise diagnostic assessment of the patient, as well as the stratification and application of a targetted therapeutic product (Fig. 2).

**Table 2:** Current precision medicine in AIT

1. Precise diagnosis with history, skin prick tests and specific IgE	
2. Proven indications: Allergic rhinitis and conjunctivitis, asthma, venom allergy	
3. Patient stratification: those who require AIT for the control of symptoms and the alteration of the natural history of allergy	
4. Innovative product: allergen standardized, evidence medicine based and with marketing authorization	
5. Placing the patient’s (and caregiver)’s wishes and goals at the centre an essential component

**Table 3:** Future precision medicine in AIT

1. Precision diagnosis aided by eHealth technologies [8, 9], enhanced Component-resolved diagnostics (CRD), genomics and other future possibilities	
2. Amenability of Allergic rhinitis, asthma, co-morbid allergic diseases, venom allergy, food allergy, other diseases (eg atopic dermatitis) to treatment with AIT	
3. Patient stratification aided by e-HEALTH assessment and biomarkers	
4. Innovative products: recombinant allergens and new forms of AIT	
5. Marketing authorization for AIT products	
6. A new role of preventative immunotherapy aimed at those at high risk	
7. Putting the patient at the centre: guideline informed rather than guideline directed.

## Review

### Immunologic rationale

Immunoglobulin E (IgE) mediated allergic diseases are characterised by heterogeneous clinical phenotypes and a large variety of different sensitisation patterns. Patients may be sensitized to few or many allergens (monoclonal, oligoclonal or polyclonal sensitization), may express various clinical organ manifestations (allergic rhinoconjunctivitis, atopic dermatitis, asthma, food allergy) with or without systemic reflection of the local IgE antibody responses, and may show different progression in terms of onset of disease, immune response patterns, organ involvement and severity of symptoms.

Treatment strategies therefore need to be individualized: from primary to tertiary prevention to organ-specific symptomatic pharmacotherapy. Furthermore, preventive or causal treatment with AIT aiming at relevant allergens should complement each other depending on the sensitization pattern of the patient and on his/her clinical presentation. Organ manifestations, severity and course of disease as well as response to allergen avoidance and pharmacological treatment and the level of control achieved by those treatments should be evaluated based on history, questionnaires and clinical investigations. Furthermore, the presence of IgE-mediated sensitization should be tested using a simple screening test based on the 4–8 most frequent allergens or allergen mixtures (Phadiatop principle) allowing for a broad application at the GP/pharmacy level. A standardized skin prick test may serve this purpose; for Europe, a panel of allergen extracts relevant to the regions has been defined [10].

With a positive skin prick test confirming that sensitization to allergens is present in an individual patient and that natural allergen exposure of the patient leads to respiratory symptoms, specific profiling of IgE sensitisation may be performed. The component-resolved molecular diagnostic tool (CRD) can be used to decipher the patient’s sensitization patterns and cross-reactivities at the molecular level [11]. CRD supports the prediction of the risk of severe reactions to specific allergens as well as the prediction of disease development in an individual patient, and thus allows an evaluation of the risk of disease comorbidities and exacerbations, and consequently appropriate risk management (risk stratification). However, there may be difficulties in the interpretation of certain CRD results [12, 13].

CRD also helps to improve the selection of the allergen product for AIT of an individual patient [14]. Ideally, IgE specificities of the patient and allergens in the product should match CRD represents a useful tool to distinguish genuine sensitisations from cross-reactions specifically in poly-sensitized patients, when traditional diagnostic tests and clinical history are unable to precisely identify the relevant allergen(s) for AIT. The approach of AIT is “precise” and tailored to the specific IgE spectrum of the individual. As AIT only modifies the immune response against the allergen for which it is being administered. a precise etiological diagnosis is required. Non specific cross-reactive binding to profilins [15] or cross-reactive carbohydrate determinants (CCDs) [16] need to be excluded, and the allergen molecule pattern, reflecting minor and major allergens, needs to be established. Therefore, the selection of an AIT product is necessarily a precise, precision approach.

In patients with sensitizations of questionable clinical relevance at the mucosal level, a target organ provocation test may be indicated before the initiation of AIT. Although not broadly available, conjunctival and nasal allergen provocation tests may confirm or reject specific allergens as causal agents of organ-specific disease.

Upon the application of the precise individualized allergen composition [17], allergen tolerance is created and induced by several mechanisms, including alterations of the allergen-specific IgE, IgG4 and IgG isotypes with the generation of blocking antibodies corresponding to the allergens in the AIT [18, 19]. Allergen tolerance also includes the induction of allergen-specific regulatory subsets of T and B cells, releasing immune-suppressive secreted factors such as IL-10 and TGF-β [20]. As a consequence, allergen-specific inflammatory responses by mast cells, basophils and eosinophils in inflamed tissues are down-regulated, leading to diminished early- and late-phase responses to the administered allergens. It is likely that mechanisms of SLIT are not identical to those of SCIT [21].

## Predictive markers of response

### Biomarkers

Biomarkers, defined as characteristics that indicate biological processes, are essential for monitoring the health of both individuals and communities. Biomarkers include physical examinations (e.g. blood pressure), biological and genetic tests, as well as others that can be objectively measured and used as indicators of pathogenic processes and changes which may occur as a result of treatment [22]. As such, they hold the potential for improving (i) the understanding of molecular mechanisms of diseases, (ii) identification of possible new disease pathways, (iii) prediction models of complex diseases, (iv) the determination of the level of biological activity of the disease, (v) refinement of disease phenotypes that may respond differently to specific treatments, (vi) the monitoring of treatment responses, and (vii) the potential application of precision medicine [23, 24]. Although much of the biomarker research in asthma and allergy to date has focused on genetics and genomics, in recent years, strategies that involve direct measurement of multiple protein concentrations have received increasing attention. Biomarkers are attractive as they may provide stronger signals of association with phenotypes of interest and because advances in biotechnology have made it possible to simultaneously measure concentrations of hundreds of candidate proteins in serum and other bio-fluids and, in turn, to investigate at the protein level the involvement of multiple pathways in disease risk [25, 26]. Biomarkers can change over time in response to a broad range of hostand environmental factors as well as treatment.

Most biomarker studies in asthma and allergic diseases have used serum or plasma samples, which are relatively easy to collect and process. Serum biomarker studies may be particularly effective in illnesses that, similar to allergic diseases, have significant systemic components and/or multi-organ manifestations [27]. Substantial progress in molecular immunology coupled with an increasing focus on translational research has resulted in a rapid expansion of immune biomarkers in recent years. These hold great promise to deliver future candidate biomarkers in immune-mediated diseases {Willis, 2015 #31843} including allergic diseases {Sorensen, 2011 #31846}.

There is a lack of biomarkers for many NCDs, their co-morbidities, disease severity and activity. This is creating a bottleneck in the discovery and development of new medicines as identified by the Innovative Medicines Initiative (IMI).

Single biomarkers and their combinations should be developed from the knowledge of mechanisms to refine and empower diagnosis (classification), find novel therapeutic approaches and better understand disease progression and prognosis (stratification). The evaluation framework of biomarkers should consist of 3 steps [28] (Table 4).

**Table 4:** Steps for the evaluation framework of biomarkers

• Analytical validation of reliability, reproducibility, and adequate sensitivity and specificity.	
• Qualification to ensure that the biomarker is associated with the clinical outcome including prognosis.	
• Utilisation analysis to determine that the biomarker is appropriate for the proposed use.	

Biomarkers in allergic diseases and asthma are of great importance and a large body of research has been started. Biomarker identification is based on systems biology approaches combining transcriptomics, proteomics, epigenetics and metabolomics in a large patient cohort. Two EU-funded projects are currently ongoing: U-BIOPRED (IMI) in severe asthma [29, 30] and MeDALL (FP7) in allergy (27, 30 ). MeDALL has already made critical observations concerning IgE biomarkers for the diagnosis and prognosis of allergic diseases [17, 31]. It is hoped that these projects will help to find biomarkers for AIT efficacy and safety, and to make precision medicine possible [32].

In AIT, 5 types of biomarkers are needed (Table 5).

**Table 5:** Types of biomarkers for AIT

1. Identification and validation of biomarkers assessing the probability of response to treatment of AIT before it is initiated	
2. Identification and validation of biomarkers assessing the safety of AIT before it is initiated	
3. Identification and validation of biomarkers confirming the efficacy of AIT in patients receiving AIT (short and long term)	
4. Identification and validation of biomarkers predicting the long-term effects of AIT before it is stopped	
5. Identification and validation of biomarkers predicting the relapse of symptoms when AIT is stopped	

### Precision medicine using e-health, integrated care pathways (ICP) and a clinical decision support system (CDSS)

Identifying the most suitable patients for whom an intervention is appropriate is critical for the delivery of a cost-effective health system. In many diseases, the management of patients uses ICT (information communication technology) tools including integrated care pathways, e-health and clinical decision support systems (CDSS). This has made a significant improvement and has sometimes led to a change of management in health systems.

Integrated Care Pathways (ICPs) differ from practice guidelines as they are utilized by a multidisciplinary team and have a focus on the quality and co-ordination of care [33]. An ICP is intended to act as a guide to treatment. Clinicians are free to exercise their own professional judgments as appropriate. However, any alteration to the practice identified within this ICP must be noted as a variance [34].

Integrated Care Pathways for asthma and rhinitis comorbidity (AIRWAYS-ICP) are multisectoral ICPs which can be used across Europe and other countries. They allow a variance analysis on the diagnosis and treatment of allergic diseases in different European countries and regions [8]. In the future, it is expected that AIRWAYS-ICPs will be linked with pay-for-performance (P4P), audit and feedback, as well as integration of recommendations with electronic medical records. AIRWAYS-ICPs place a particular interest in cultural and societal aspects of the diseases in a project centred around the patient. AIRWAYS ICPs represent the model of NCD used by the European Innovation Partnership on Active and Healthy Ageing [35] and will be used as a model for deployment and scale up of chronic diseases by the EIP on AHA.

Telemonitoring permits the monitoring of patients on a regular basis. Pollen allergy using electronic data management of sensitized patients has been proposed by the European Allergy Network (EAN) (www.pollenwarndienst.com). The allergy sentinel network, approved by AIRWAYS-ICPs, is a very simple iPhone/Android App which is already available and which is being expanded to other systems with interoperability. It is initially being deployed in 13 different countries with 14 languages. Current status is measured daily by the use of VAS ( Visual Analogue Scales) which represent reliable and valid measures of rhinitis control [36, 37]. It can be used across the life cycle [38–40]. Daily, 4 VAS (global evaluation, nasal, ocular and bronchial symptoms) are filled in by the patient on a cell phone and the information is sent to a clinical decision support system (CDSS) for an optimal management of the patients.

The clinical decision support system (CDSS), an interactive computer software, is designed to assist health professionals with decision making tasks, such as determining treatment strategies of patient using the results of ICPs. This knowledge management provides clinical advice for patient care. The chronic respiratory diseases CDSS (AIRWAYS-CDSS) will be based on the ARIA 2015 revision (in preparation) to allow standardisation of patient management. It will also enable patient stratification. Patients with uncontrolled disease based on VAS telemonitoring despite optimal treatment according to guidelines will be considered as SCUAD (severe chronic upper airway diseases) [41]. However, However the dialogue to date suggests that this sis a support tool for patients and that the CDSS will prompt the patient concerning different treatment options.

These 3 innovative tools (AIRWAYS-ICP, allergy sentinel network and AIRWAYS-CDSS) will be combined in the MACVIA-ARIA Sentinel NetworK (MASK) and will make it possible to assess some of the unmet research needs in AIT (Table 6).

**Table 6:** Precision medicine in AIT using innovative tools (biomarkers and e-health)

• Assessment of prevalence and severity of allergic diseases.	
• Phenotypic characterisation of allergic patients, stratification of patients, characterisation of severe chronic upper airway disease (SCUAD) patients and characterisation of patients to be treated by AIT.	
• Randomised controlled trials (placebo-controlled or real life cluster randomised trials): assessment of efficacy (during the allergen exposure) and safety (during AIT administration).	
• Follow up of patients in clinical settings during AIT.	
• Follow up of patients in clinical settings after AIT has been stopped.	

These tools can provide valuable information on most parameters for randomized controlled trials (RCTs) in AIT [42] (Table 7).

**Table 7:** Use of biomarkers and e-health in AIT randomized controlled trials

EAACI AIT outcomes	e-health and biomarkers
Total symptom score	Included in VAS^a^
Total medication score	Standardised by ICPs and CDSS
Total symptom-medication score	Standardised by ICPs and CDSS
Quality-of-Life	Included in VAS^a^
Well days	Standardised by ICPs and CDSS
Days with severe symptoms	Standardised by ICPs and CDSS
Global assessment of patient satisfaction	Recorded by telemedicine

^a^In 2 cluster randomized trials of 595 and 537 patients (one in secondary care, one in primary care) [43, 44], the VAS level change during treatment incorporated symptom and RQLQ information.

### Biomarkers and e-health in preschool children

To date, AIT has only been considered after 5 years of age in most recommendations, but newer ones will advocate this form of treatment in highly selected children from 3 years of age. Pre-school children who may be candidates for SLIT are those sensitized to indoor allergens (mites) with co-morbid asthma and rhinitis [45–47] and possibly those with polysensitization [31] or pollen allergic children with demonstrated symptoms during the season. It is likely that CRD will be of great value in the identification and stratification of preschool children who might benefit from early AIT. VAS can be used satisfactorily by caregivers of preschool children [39] with e-health providing an innovative tool for monitoring outcomes..

The combination of biomarkers with MASK will represent a novel tool to diagnose, stratify, manage and assess treatment efficacy in allergic patients.

## Understanding the economic burden of uncontrolled and under-treated allergic respiratory disease (allergic rhinitis and asthma)

Respiratory diseases place a huge burden on society in terms of disability and premature mortality. They also have an impact on direct costs (health service and drugs prescribed) and on indirect costs related to lost production.

### Allergic rhinitis

Allergic rhinitis is one of the most common diseases globally, in all age groups and in all countries. Many patients do not seek medical care because the disease is often underestimated or unrecognised by patients as well as by health care professionals.

Patients may not understand the benefits of treatment and compliance to treatment may then be poor. A substantial proportion of patients can be managed by optimal pharmacological treatment [1]. However, a subset of patients (10 to 20 %) is poorly controlled and is ascribed to SCUAD (severe chronic upper airway disease) (41, 48, 49).

Asthma is often associated with allergy and it is well known that severe asthma has an important impact on the health system [48- 51].

Allergists and their patients know that the symptoms of allergic diseases can disturb sleep, worsen mood, decrease energy levels, impair quality of life and weaken the ability to concentrate. In children, these factors impact performance at school [52], potentially frustrating ambitions for higher education and impairing active and healthy ageing [53]. In the work environment, impaired concentration leads to decreased productivity, a phenomenon recognised as presenteeism, which has a consequent negative economic impact. The patient also suffers an economic burden. Multiple visits to the physician, multiple prescription costs and potentially the costs of side effects of medications, particularly when first generation antihistamines [9] are prescribed, all constitute a significant amount of direct cost. Time off work due to absenteeism as well as reduced performance at work also represent an economic burden to the patient.

A recent GA^2^LEN review limited to the European Union attempted to assess the costs of allergic diseases. Although the study was carefully performed, it did not include a sufficient sensitivity analysis with the resulting published costs (between 55 and 151 billion € per year) coming in at the higher levels of expectation [54]. This study rightly pointed out that there is an urgent need to treat patients appropriately, an achievement which would likely reduce indirect costs.

### Asthma

The prevalence of asthma in developed countries is around 10 % [55] with large differences between countries. Prevalence in emerging countries is sometime lower but continues to rise sharply with increasing urbanization and westernization [56, 57]. It is estimated that 300 million people suffer from asthma globally [58].

In 2012, the European Respiratory Society published a white book [59, 60]. In asthma, the annual cost of health care and lost productivity is about €34 billion in the 28 countries of the EU. The average direct healthcare cost per case in asthma is about €2000 per year, often over several decades. The value of Disability-adjusted life-years (DALYs) lost is €38 billion in asthma leading to a total cost (direct + indirect + DALYs) of about €72 billion in the EU [59, 60].

### Allergen immunotherapy is a cost effective treatment

Cox and Hankin reviewed studies from health systems in North America and in southern, central and northern Europe, in which AIT and symptomatic drug therapy were used to treat a range of perennial and seasonal allergic conditions [61]. Twenty-four studies met their inclusion criteria, and all but one of the studies concluded that AIT was associated with cost savings (relative to symptomatic drug therapy). The savings range from a few hundred dollars/euros to a few thousand per year. The only study which failed to demonstrate savings for AIT was a double-blind, randomized, placebo-controlled trial published in 1996, which was limited in that it only compared the costs of medication for the first two years of SCIT (relative to standard treatment) in 77 adolescents and adults with ragweed-pollen-induced asthma [62]. However, many of these studies were based on assumptions of the preventive effect of AIT using prediction models.

Nine studies looked at the health economics of SCIT, eight looked at SLIT tablet formulations, two covered liquid SLIT products, four compared the health economics of SLIT, SCIT and standard treatment and one compared the health economics of SCIT and SLIT [63]. A variety of models were used: comparative cost analysis, a decision-tree cost model, a Markov model and an analysis of the savings associated with symptom-free days. Of the six studies comparing SLIT with SCIT, four found cost savings for SLIT and two found cost savings for SCIT. In a recently published study [64], a 5-grass pollen SLIT tablet administered with a pre- and co-seasonal regimen was associated with significant cost savings compared with year-round SCIT ($2471), seasonal SCIT ($948) and a single-grass pollen SLIT tablet administered year-round ($1168).

A health technology assessment examined the comparative costs of SLIT and SCIT. Benefit from both SCIT and SLIT compared with placebo was consistently demonstrated, but the extent of this effectiveness in terms of clinical benefit is as yet unclear [65]. Both SCIT and SLIT may be cost-effective compared with SDT from around 6 years (threshold of £20,000-30,000 per QALY). The authors concluded that further research is needed to establish the comparative effectiveness of SCIT compared with SLIT and to provide more robust cost-effectiveness estimates.

Given the relatively high numbers needed to treat (to obtain benefit) and low numbers needed to harm, it becomes more attractive to consider a strategy which fundamentally alters the disease trajectory. Of equal importance is improving patient selection which should reduce the numbers needed to treat to see benefit, thus substantially reducing overall costs.

Over the life course of the individual, the picture of AIT becomes more attractive economically given the demonstrated societal economic benefits, the reduced healthcare costs and the reduction in economic costs to the individual patient.

## Concluding remarks

The aim of this article was to review and position AIT in the context of Precision Medicine.

AIT has unique immunological rationale, since the approach is tailored to the specific IgE spectrum of an individual and modifies the natural course of the disease as it demonstrates persistent efficacy after cessation of active treatment.

Precise information and biomarkers provided by systems medicine [32] and network medicine [66] will address the discovery of AIT biomarkers for (i) precise identification of aetiology, which leads to (ii) stratification of patients eligible for AIT ( i.e. patient selection) and (iii) assessment of AIT efficacy.

This area of medical technology is evolving rapidly with e-health influencing the way we practice medicine. Biomarkers associated with e-health and a clinical decision support system will change the scope of AIT. It will help to monitor the patient’s control and will provide data for (i) patient stratification, (ii) clinical trials, (iii) monitoring the efficacy and safety of targeted therapies, all of which are critical components for achieving appropriate reimbursement.

The cost/effectiveness of AIT is a key issue for patients and purchasers. It should include long-term effects and benefits in the pharmaco-economic evaluation, since no other current allergy treatment has this specific characteristic of disease modification. Similarly, the costs of potential harms, in particular due to side effects and lost productivity, associated with current treatment modalities need to be factored in.

### The challenge: stratification of the patient requiring AIT

AIT can be initiated in two different situations that need different considerations and to close different research/therapeutic gaps:

- Identification of the patient most likely to benefit from early intervention (prevention strategy). The prevention of allergic diseases by the identification of children who have developed asymptomatic sensitization and early commencement of immunotherapy is a hot topic but early results are far from conclusive [67].
- The patient with severe uncontrolled disease (treatment strategy). The subject of precision medicine is of course the individual patient. Impairment of quality of life with allergic rhinitis is greater than that caused by asthma [68]. The negative impact of allergic diseases is not confined to the patient alone: the activities of the whole family, especially social, recreational and sporting, may be governed by the need to avoid going outside, for example. The immunologic and clinical responses of allergic patients with severe disease and multiple sensitisations need to be better understood in terms of mechanisms, trajectories of diseases [31] and response to AIT. The importance of comorbidities and polysensitization merits further investigation to improve our understanding of this complex biodynamic. It is likely that a scoring system will be developed combining risk factors such as family history and early manifestation of allergic disease coupled with evidence of sensitisation by either skin prick test of specific IgE or other biomarkers.This could identify the population for whom such an approach might be beneficial.

**Fig. 3:**
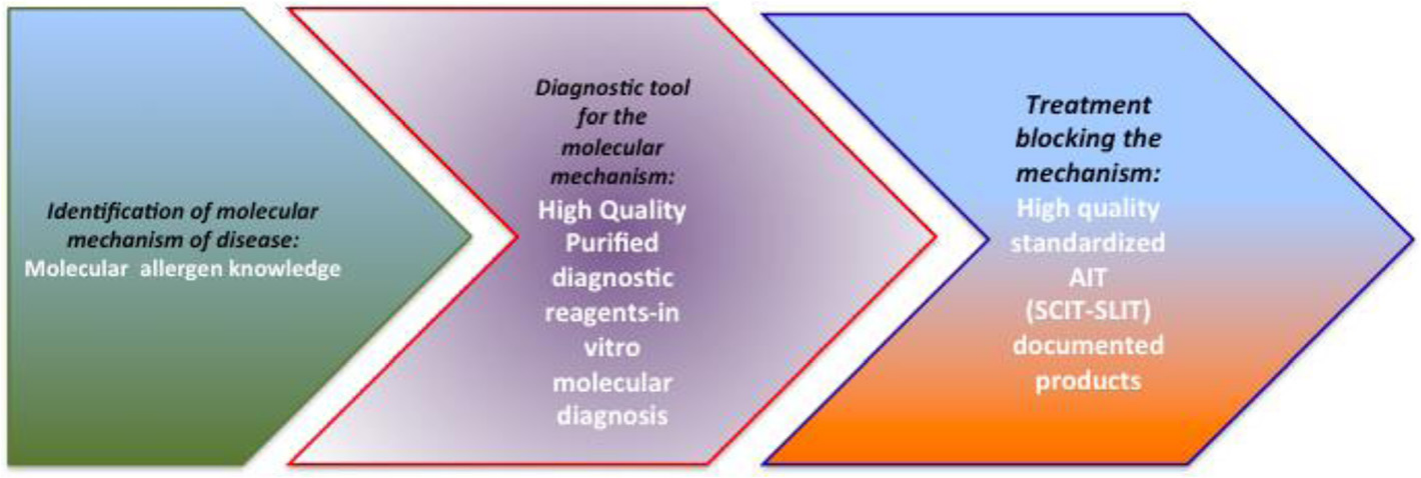
Critical issues on precision medicine in AIT

**Fig. 4:**
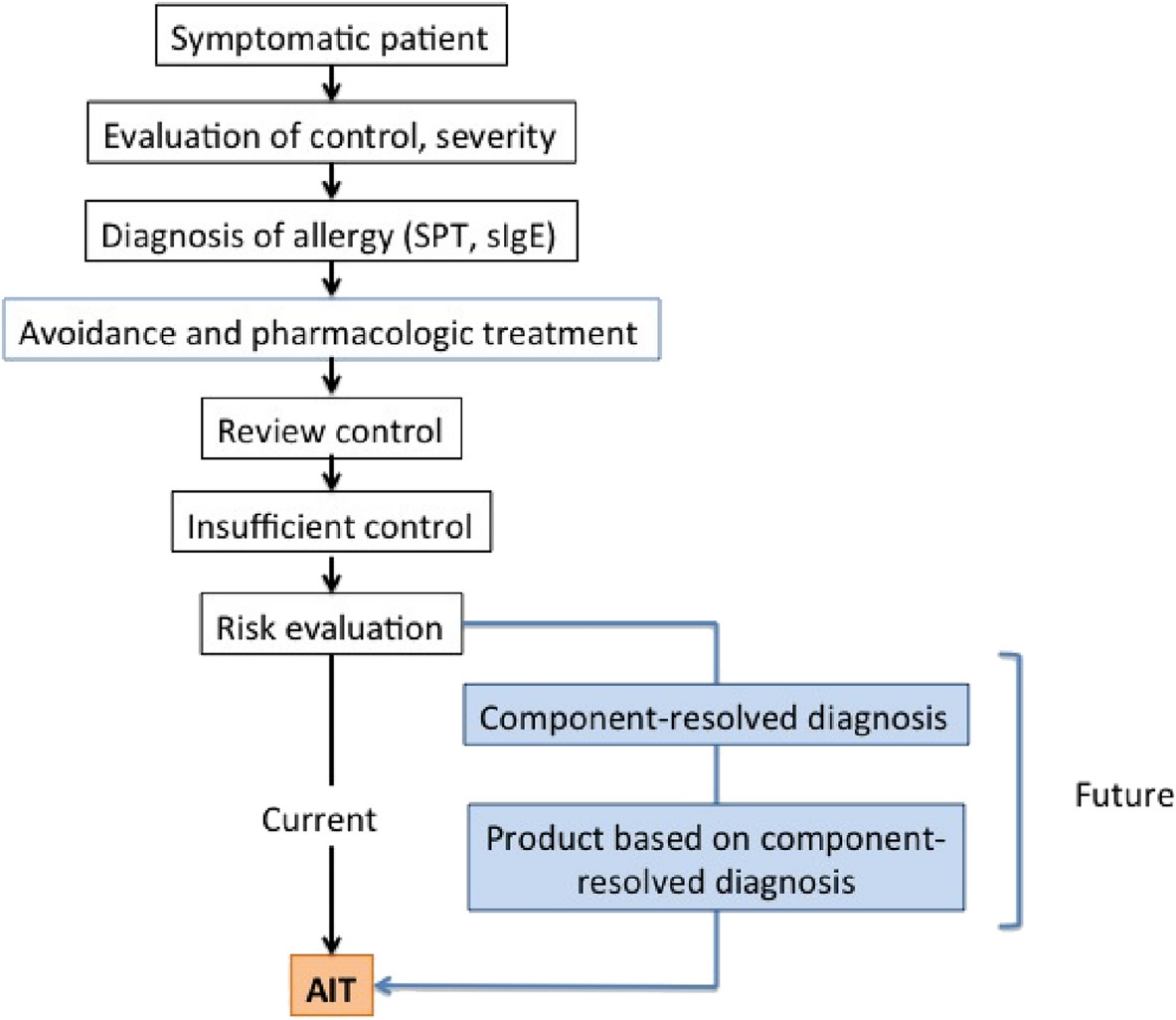
Flow of precision medicine approach in allergic disease

Precision medicine is therefore a paradigm for current and future AIT (Fig. 3 and 4). Partnerships between the allergy scientific community, manufacturers, all other stakeholders in the healthcare system, politicians and economists should be promoted and strengthened to change the paradigm of AIT in medicine.

## Abbreviations

AIRWAYS ICPs: Integrated Care Pathways for Airway Diseases
AIT: Allergen immunotherapy
ARIA: Allergic Rhinitis and its Impact on Asthma
CDSS: Clinical decision support system
CRD: component-resolved molecular diagnostic
EIP on AHA: European Innovation Partnership on Active and Healthy Ageing
GA^2^LEN: Global Allergy and Asthma European Network
ICP: Integrated care pathway
MACVIA: Contre les MAladies Chroniques pour un VIeillissement Actif
MASK: MACVIA-ARIA Sentinel NetworK
MeDALL: Mechanisms of the Development of Allergy (FP7)
NCD: non communicable disease
SCIT: Sub-cutaneous immunotherapy
SLIT: Sublingual immunotherapy
SCUAD: Severe Chronic Upper Airway Disease
U-BIOPRED: (IMI)
VAS: Visual analogue scale
